# Subtoxic cell responses to silica particles with different size and shape

**DOI:** 10.1038/s41598-020-78550-5

**Published:** 2020-12-09

**Authors:** Markus Kersting, Mateusz Olejnik, Nina Rosenkranz, Kateryna Loza, Marina Breisch, Alexander Rostek, Götz Westphal, Jürgen Bünger, Nadine Ziegler, Alfred Ludwig, Manfred Köller, Christina Sengstock, Matthias Epple

**Affiliations:** 1 BG University Hospital Bergmannsheil, Surgical Research, Ruhr University Bochum, Bochum, Germany; 2 Inorganic Chemistry and Center for Nanointegration Duisburg-Essen (CeNIDE), University of Duisburg-Essen, Essen, Germany; 3 Institute for Prevention and Occupational Medicine of the German Social Accident Insurance, Institute of the Ruhr University Bochum (IPA), Bochum, Germany; 4 Chair for Materials Discovery and Interfaces, Institute for Materials, Faculty of Mechanical Engineering, Ruhr University Bochum, Bochum, Germany

**Keywords:** Environmental sciences, Nanoscience and technology

## Abstract

Health risks from particles are a priority challenge to health protection at work. Despite the ubiquitous exposure to a wide range of particles and the many years of research in this field, there are fundamental unresolved questions regarding the prevention of particle-related respiratory diseases. Here, the highly relevant particulate material silicon dioxide was analyzed with emphasis on defined size and shape. Silica particles were prepared with different size and shape: Spheres (NS nanospheres 60 nm; SMS submicrospheres 230 nm; MS microspheres 430 nm) and rods (SMR submicrorods with *d* = 125 nm, *L* = 230 nm; aspect ratio 1:1.8; MR microrods with *d* = 100 nm, *L* = 600 nm; aspect ratio 1:6). After an in-depth physicochemical characterization, their effects on NR8383 alveolar macrophages were investigated. The particles were X-ray amorphous, well dispersed, and not agglomerated. Toxic effects were only observed at high concentrations, i.e. ≥ 200 µg mL^−1^, with the microparticles showing a stronger significant effect on toxicity (MS≈MR > SMR≈SMS≈NS) than the nanoparticles. Special attention was directed to effects in the subtoxic range (less than 50% cell death compared to untreated cells), i.e. below 100 µg mL^−1^ where chronic health effects may be expected. All particles were readily taken up by NR8383 cells within a few hours and mainly found associated with endolysosomes. At subtoxic levels, neither particle type induced strongly adverse effects, as probed by viability tests, detection of reactive oxygen species (ROS), protein microarrays, and cytokine release (IL-1β, GDF-15, TNF-α, CXCL1). In the particle-induced cell migration assay (PICMA) with leukocytes (dHL-60 cells) and in cytokine release assays, only small effects were seen. In conclusion, at subtoxic concentrations, where chronic health effects may be expected, neither size and nor shape of the synthesized chemically identical silica particles showed harmful cell-biological effects.

## Introduction

Silica is the oxide of silicon, i.e. SiO_2_. If prepared by wet-chemical synthesis, it is usually X-ray amorphous and can be represented by the general formula SiO_2_·x H_2_O (amorphous silica or polysilicic acid). Several anhydrous and crystalline forms are also known, with quartz (stoichiometric SiO_2_) being the most prominent^1^. In combination with other metal oxides, crystalline silicates form the biggest part of the earth's crust, and are almost ubiquitous. Silica particles (usually in the X-ray amorphous form) have many applications, e.g. in food or consumer products, as well as in biomedicine for potential therapeutic applications like gene and protein delivery and optical imaging^2−4^. Due to favorable properties like colloidal and chemical stability, the possibility for a covalent surface modification, and a high specific surface area, the application cases for silica particles and silica-based materials have grown rapidly^5−8^. Still, the development and synthesis of silica particles with precisely controlled physicochemical properties like shape, particle size distribution, and surface charge are critical steps for their toxicological evaluation and also for their use for biomedical application^7,9−17^.

Silica (nano-)particles are also of high importance in food and consumer products^1,18^. Although silica is considered to be non-toxic after oral intake^18^, it can cause inflammatory effects after inhalation^6,19−22^. Continuously high levels of inhalative particle exposure, including silica, lead to a chronic inflammation of the lung, characterized by the accumulation of inflammatory cells, in particular alveolar macrophages and neutrophil granulocytes^23^. Chronic inflammation can lead to fatal lung dysfunction, including chronic obstructive pulmonary disease (COPD), fibrosis (such as silicosis and asbestosis), and cancer^21,24−30^. Crystalline silicon dioxide (like quartz) appears to be more harmful than amorphous silica, possibly due to its different surface nature and lower water content^8,18,21,29,31^.

Although there is extensive literature on the biological effects of silica particles, most studies are difficult to compare because of different particle properties like size, shape, charge or surface functionalization^27,32^. Usually, the cell types challenged with silica particles are also different which makes a comparison difficult^14,18,27,33^. Thus, there is no consensus about the different and potentially adverse biological effects of silica^26^. Furthermore, only few methods have been described for the preparation of monodisperse rod-shaped silica particles without templates or polymer additives, being present in well-dispersed and not agglomerated form.

Thus, our aim was to prepare silica particles with different shape (spheres and rods) and different size from nano- to microscale, but with identical surface chemistry. Five morphologically different but chemically uniform silica samples were synthesized and characterized, i.e. all parameters were kept constant except for particle size and shape. Special emphasis was laid on the inflammatory potential in the subtoxic concentration range (particle concentration which provoked less than 50% cell death compared to the untreated cell control) which is less frequently investigated, but may have strong effects in a persistent long-term exposition, e.g. promote a chronic inflammation. As the chemo-attraction of neutrophils is a key event in inflammation, the particle-induced migration of neutrophils is suggested as a functional and predictive in vitro model for the mechanistic investigation of a particle-induced inflammation of the lung^34−36^.

## Results

The first synthesis of monodisperse spherical silica particles with a narrow size distribution was reported by Stöber et al.^37^. Based on this method, many modifications to synthesize silica particles in the size range of 50 nm to 1 µm by the hydrolysis of tetraethyl orthosilicate (TEOS) in the presence of ammonia have been published. Particle size and shape can be adjusted by the choice of additives^5,9,38^. We prepared spherical particles with three different diameters (denoted here as nanospheres NS: 60 nm, submicrospheres SMS: 230 nm, and microspheres MS: 430 nm) by variations of the Stöber method^39^. The particle size was controlled by changing the molar ratios of the reagents in the reaction mixture (TEOS, water and ammonia) and performing syntheses at different temperatures (25 °C, 47 °C, 68 °C). For the synthesis of rod-shaped silica particles with different lengths (denoted here as submicrorods SMR: 125 nm·230 nm, and as microrods MR: 100 nm·600 nm), the cationic surfactant CTAB was used to induce a rod-shaped growth of the particles by modification of the Stöber process using the soft-template strategy^39,40^. We classified the particles as "nano", "submicro", and "micro" for the sake of simplicity for the following discussion. For microscopic cell uptake studies, all synthesized silica particles were fluorescently labeled with fluorescein isothiocyanate-functionalized polyethyleneimine (PEI-FITC). All syntheses were multiply carried out and very robust in terms of reproducibility.

The particles were characterized by scanning electron microscopy (SEM), dynamic light scattering (DLS), UV/vis spectroscopy, infrared (IR) spectroscopy and X-ray powder diffraction (XRD). All physicochemical data are summarized in Table 1.

**Table 1:** Physicochemical characterization data of the silica particles, both unfunctionalized and coated with polyethyleneimine (PEI).

	Size by SEM/nm	Size by DLS/nm	PDI from DLS	Zeta potential by DLS/mV	Size by DLS/nm	PDI from DLS	Zeta potential by DLS/mV	Particle number per g solid	Specific surface area/m^2^ g^−1^
	Unfunctionalized				Coated with PEI				
Nanospheres	60	117	0.22	− 16	222	0.32	+ 29	4.42 × 10^15^	50
Submicrospheres	230	272	0.08	− 53	360	0.21	+ 36	7.85 × 10^13^	13
Submicrorods	125·230	357	0.23	− 29	423	0.38	+ 22	2.77 × 10^14^	22
Microspheres	430	519	0.15	− 28	582	0.19	+ 39	1.20 × 10^13^	7.0
Microrods	100·600	439	0.32	− 22	564	0.45	+ 28	1.06 × 10^14^	21

All particles were completely X-ray amorphous. The particle numbers and the specific surface area were computed from the SEM particle size data. DLS was performed in water at pH 7.

**Figure 1:**
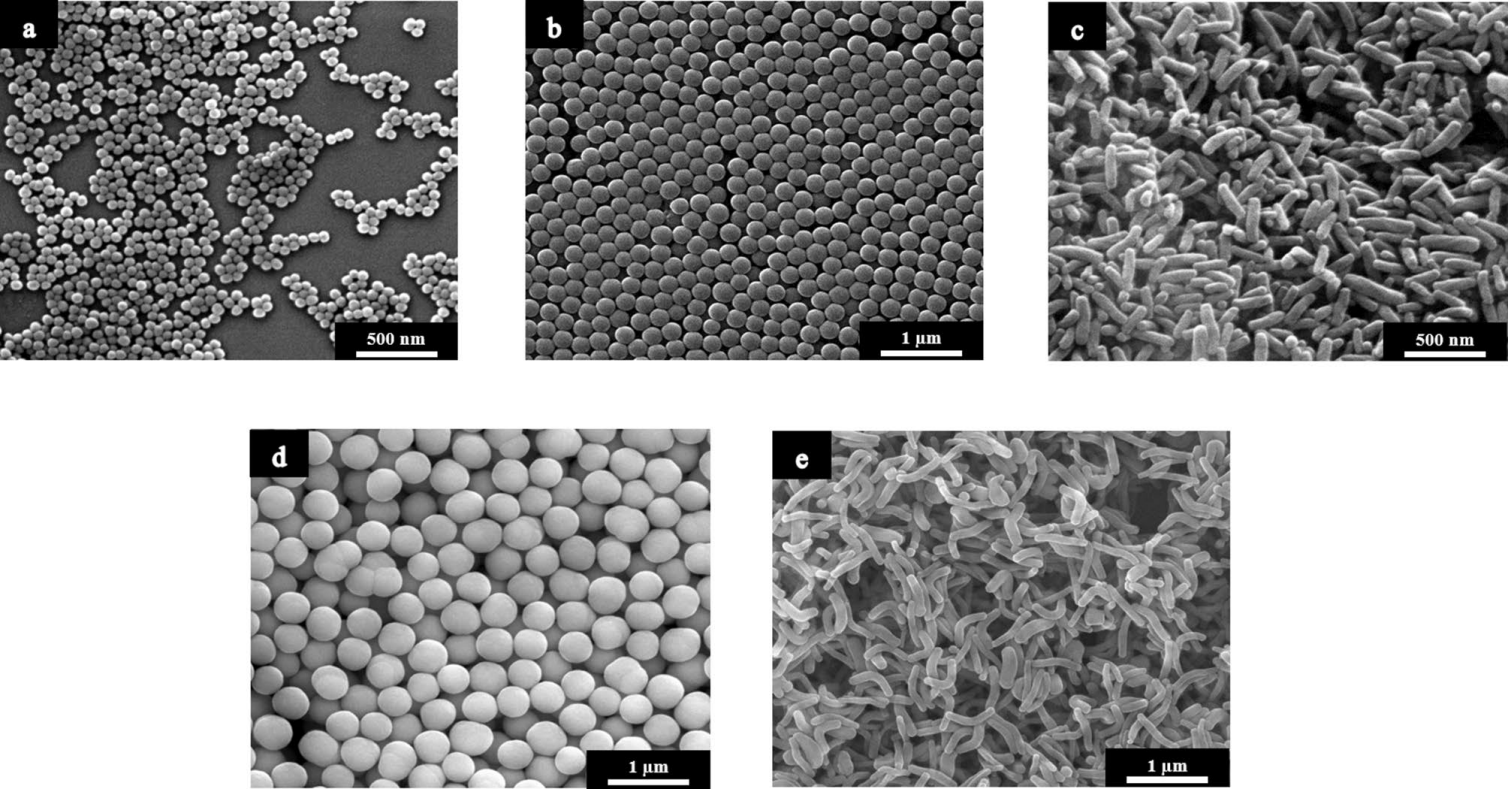
SEM images of unfunctionalized silica particles: Nanospheres (**a**), submicrospheres (**b**), submicrorods (**c**), microspheres (**d**), microrods (**e**).

**Figure 2:**
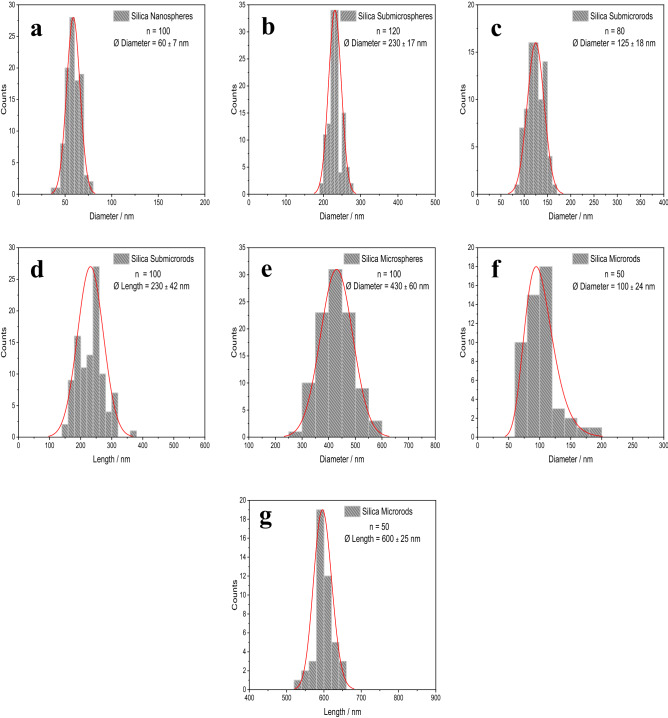
Particle size distribution (LogNormal) by SEM analysis for all unfunctionalized silica particles: Diameter of nanospheres (**a**), diameter of submicrospheres (**b**), diameter of submicrorods (**c**), length of submicrorods (**d**), diameter of microspheres (**e**), diameter of microrods (**f**), length of microrods (**g**).

The size of the silica particles and their morphology were determined by SEM (Fig. 1). Scanning electron micrographs showed spherical and rod-like particles with a narrow particle size distribution. The corresponding histograms of the number-weighted particle diameters and lengths are shown in Fig. 2. From these data, geometric parameters and particle concentrations can be derived.

**Figure 3:**
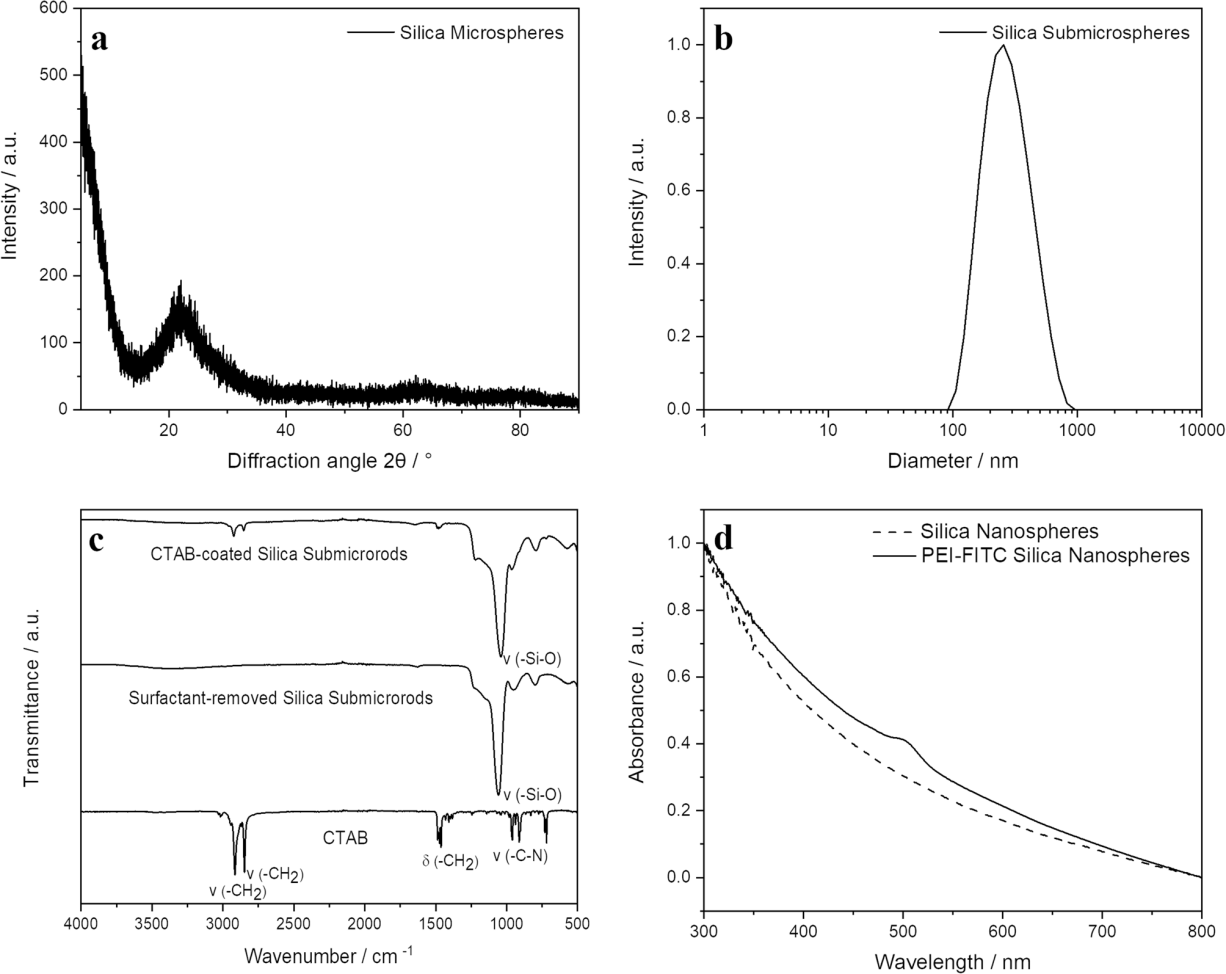
Representative characterization data of unfunctionalized silica particles (**a**–**d**) and for fluorescent silica particles (**d**): XRD shows amorphous particles (**a**), DLS shows well-dispersed particles (**b**), IR spectroscopy confirms the removal of CTAB after the synthesis (**c**), and UV/vis spectroscopy confirms the attachment of PEI-FITC (**d**). The particles with other morphologies had very similar properties.

To investigate the colloidal stability and the particle size distribution, we performed DLS measurements (Fig. 3). The polydispersity index (PDI) was below 0.3 for all particles, indicating a low degree of agglomeration in the particle dispersions^13^. This is supported by the fact that the hydrodynamic diameter by DLS was only a little larger than the diameter of the solid core as determined by SEM. The zeta potential measurements at pH 7 in water showed that all unfunctionalized particles were negatively charged (due to deprotonation of surface silanol groups) whereas all PEI-coated particles carried a positive charge due to the cationic polyelectrolyte PEI.

X-ray powder diffraction showed that all silica particles were non-crystalline, i.e. fully amorphous (Fig. 3). Thus, we can exclude the presence of crystalline SiO_2_, i.e. the particles consist exclusively of hydrated silica in the form of SiO_2_·x H_2_O. The IR spectra showed characteristic Si–O stretching bands at 1082 to 1114 cm^−1^^41^. For rod-like particles, the complete removal of CTAB (a potentially cytotoxic synthesis additive) was confirmed by IR spectroscopy (absence of C-H bands). For PEI-FITC-labeled particles, the UV/vis spectrum showed the expected absorption band at 495 nm (FITC).

**Figure 4:**
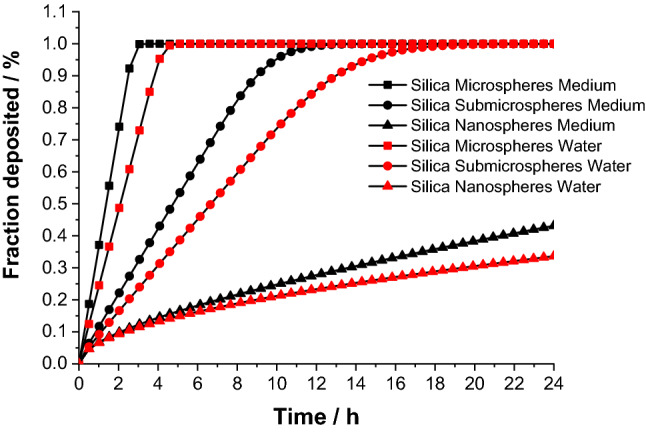
Precipitation and diffusion of spherical silica particles according to the ISDD model in water and in cell culture medium (DMEM supplemented with 10% FCS).

The cell-to-particle contact plays a strong role for the biological effects^42,43^. When it comes to contact with cells, the colloidal stability (sedimentation rate) is of prime importance. We have therefore used an in vitro sedimentation, diffusion and dosimetry model (ISDD) to estimate the particle precipitation and diffusion both in water and in cell culture media (Fig. 4)^44^. Due to their high density, the microparticles will sediment rapidly onto the cells, whereas the nanoparticles remain in dispersion for a much longer time. Table 2 summarizes the concentration data at the subtoxic concentration of 100 µg mL^−1^.

**Table 2:** Dose data for all cell culture studies with silica particles (100 µg mL^−1^) in a 24-well plate (2 cm^2^, 640 µL, 48,000 cells).

	Silica (SiO_2_) concentration/mmol L^−1^	Silica particle concentration/L^−1^	Silica particle surface/m^2^ per particle	Total silica particle surface/m^2^ L^−1^	Total silica particle surface per well (640 µL)/m^2^	Total silica particle surface per cell (48,000 cells)/µm^2^	Total silica concentration after complete sedimentation in the well/particles m^−2^
Nanospheres	1.7	4.42 × 10^14^	1.13 × 10^−14^	5.0	3.2 × 10^−3^	67,000	1.41 × 10^15^
Submicrospheres	1.7	7.85 × 10^12^	1.66 × 10^−13^	1.3	8.3 × 10^−4^	17,000	2.51 × 10^13^
Submicrorods	1.7	2.77 × 10^13^	8.01 × 10^−14^	2.2	1.4 × 10^−3^	29,000	8.86 × 10^13^
Microspheres	1.7	1.20 × 10^12^	5.81 × 10^−13^	0.70	4.5 × 10^−4^	9,300	3.84 × 10^12^
Microrods	1.7	1.06 × 10^13^	1.96 × 10^−13^	2.1	1.3 × 10^−3^	28,000	3.40 × 10^13^

As the cytotoxic effects of particles have been related to their total surface area, also with respect to the number of cells, the data were converted to different ratios (see discussion).

**Figure 5:**
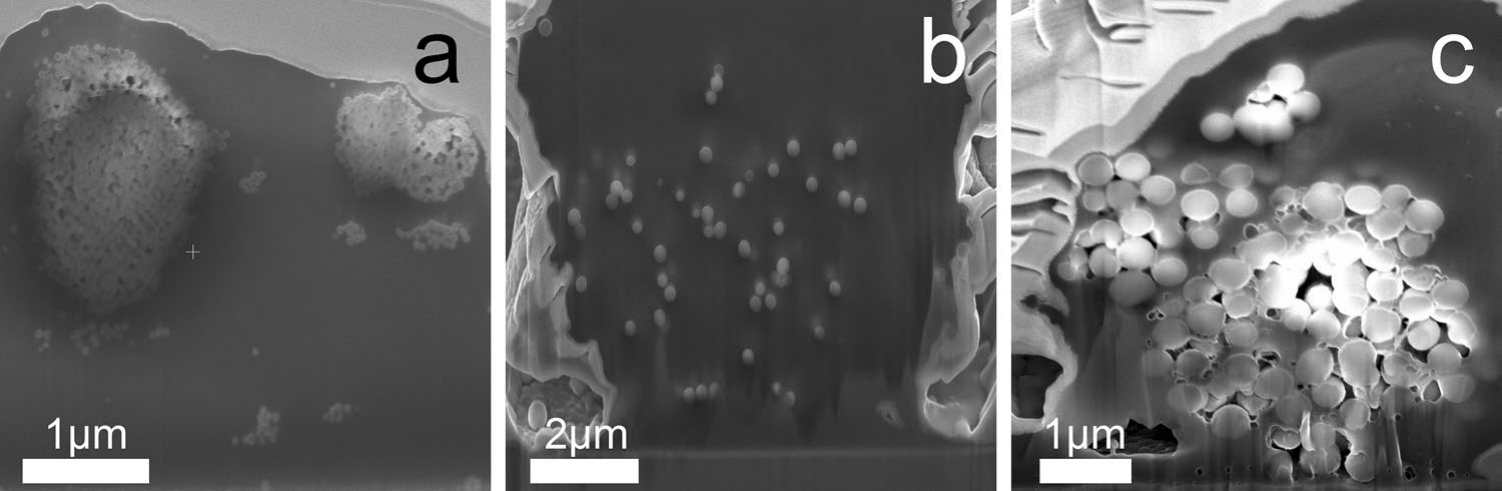
Representative SEM images of unfunctionalized silica particles in dissected (FIB milling) NR8383 alveolar macrophages after uptake (24 h; 100 µg mL^−1^): nanospheres (**a**), submicrospheres (**b**), microspheres (**c**).

As typical cells of the immune system, NR8383 rat alveolar macrophages were used for cell culture experiments and incubated with silica particles in defined doses with different specific particle surface areas. These cells have also been proposed as models for particles to elucidate inhalation toxicity in in vitro assays^32^. The activity of particles in biological systems essentially depends on whether the particles can penetrate cells as well as on their subsequent intracellular fate. In general, particles can enter cells by various pathways which can be analyzed by different methods, such as confocal laser scanning microscopy (CLSM), fluorescence-activated cell sorting (FACS), or focused ion beam milling/scanning electron microscopy (FIB/SEM), depending on the cell type and the physicochemical properties of the nanoparticle^43,45^. The uptake of unfunctionalized silica particles by NR8383 macrophages as relevant cell type in particle contact^23,27^. After 24 h of incubation was analyzed in detail using cross-sectional analysis of single cells by FIB/SEM and subsequent TEM/EDX of FIB-prepared cell lamellae. Figure 5 shows representative SEM images after FIB cutting of macrophages incubated with spherical silica nanoparticles, submicroparticles, and microparticles. The particles were found as electron-dense structures inside the cells. Notably, neither size nor shape of the nanoparticles were changed by the uptake, however, the nanospheres were mainly present as larger agglomerates.

**Figure 6:**
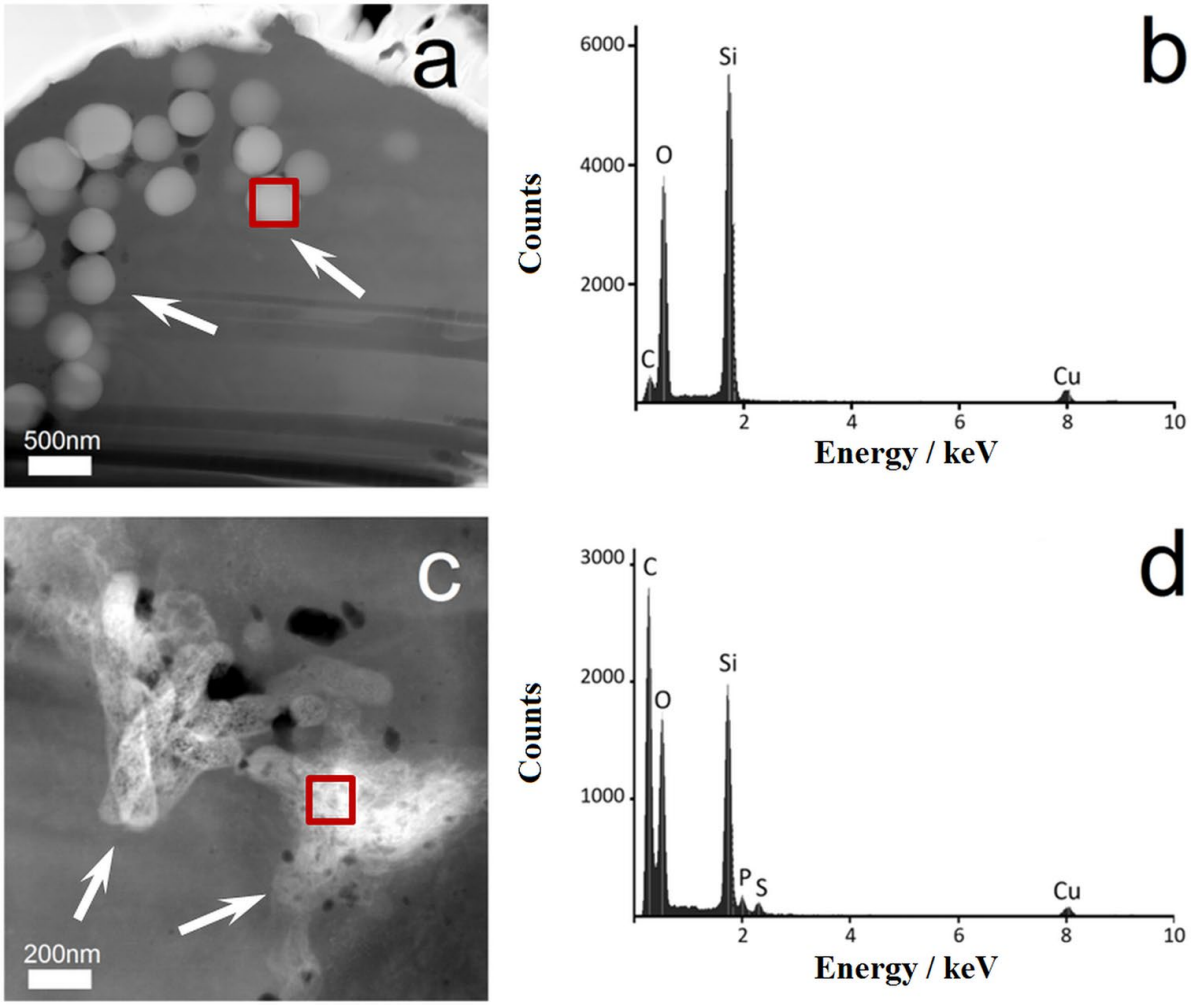
Representative TEM/EDX analyses of FIB-prepared cell lamellae from single NR8383 cells after 24 h of exposure to 100 µg mL^−1^ unfunctionalized silica particles. The thickness of each TEM lamella was 100 nm. TEM images of cell lamellae were prepared after incubation with microspheres (**a**) or microrods (**c**); white arrows indicate particles inside the cells. Corresponding EDX spectra (**b** and **d**) of the elements detected within the regions of interest of the cell lamellae (red boxes in **a** and **c**) confirmed the presence of silica.

The chemical identity of the nanoparticles after uptake was demonstrated by TEM and corresponding EDX spectra of the cells (Fig. 6). EDX spectra showed signals for carbon (C), oxygen (O), and silicon (Si). The elements C and O were detected in all organic samples due to the presence of organic cell material as shown earlier for untreated hMSC (no exposure to particles)^46^. However, the silicon signal clearly confirms the presence of silica particles. As it is generally accepted, particles are taken up by mammalian cells by endo- and pinocytosis, depending on the cell type as well as on the particle properties, i.e. size, charge (zeta potential), shape, and functionalization^43,47−53^. Typically, particles are localized as agglomerates in membrane-enclosed vesicles within the cells. For silica nanoparticles of spherical and rod-like shape it has been shown that they were all taken up by macrophages, although the endocytosis pathway was dependent on the particle geometry^54^.

**Figure 7:**
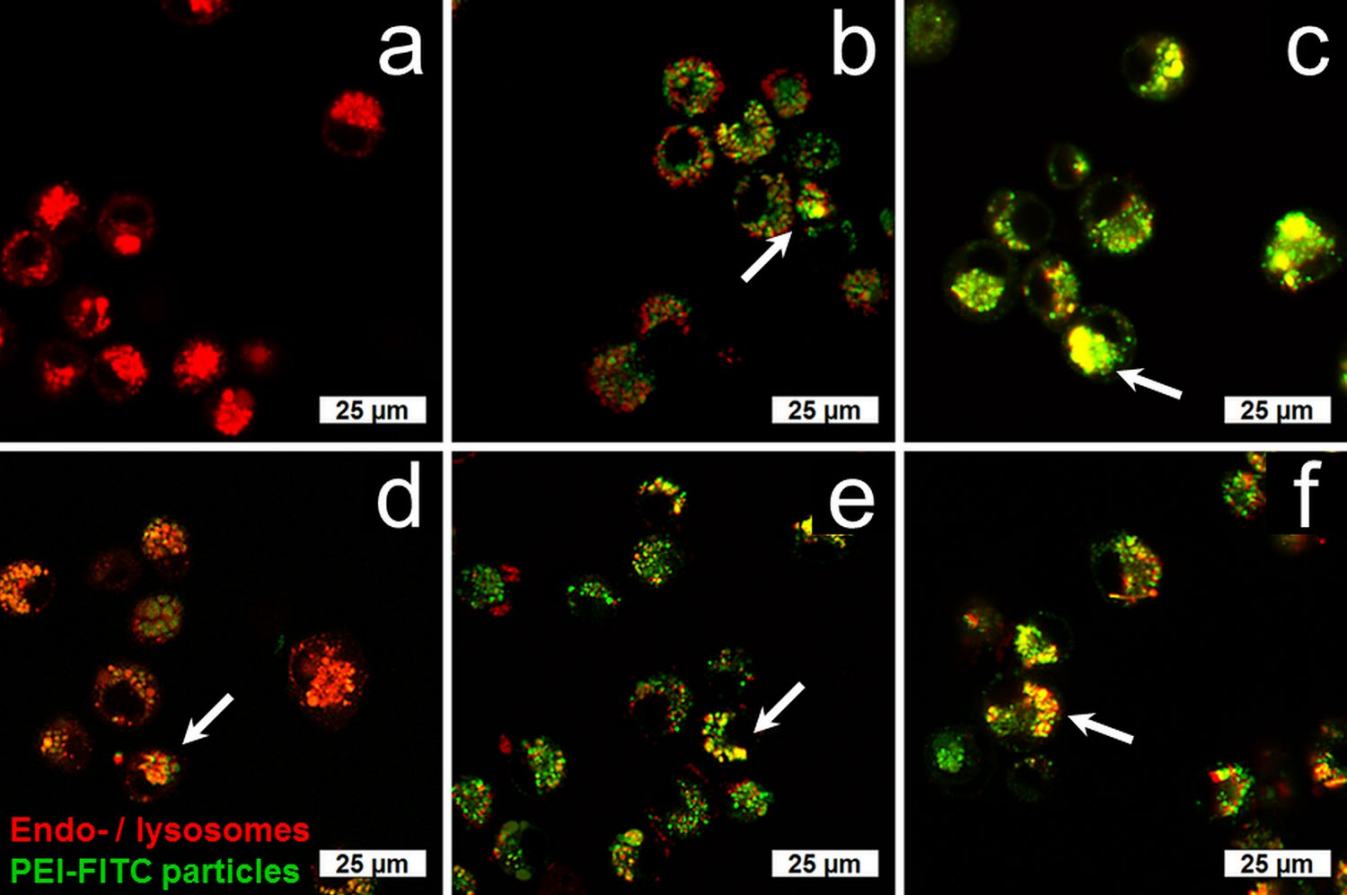
Localization of silica particles in NR8383 alveolar macrophages after incubation with PEI-FITC-labeled silica particles (100 µg mL^−1^) for 24 h by confocal laser scanning microscopy (CLSM). The red fluorescence signals represent endo-/lysosomes stained by LysoTracker Red DND-99. The green signals represent PEI-FITC-labeled silica particles. The overlay of both signals (yellow, indicated by white arrows) indicates a co-localization of particles in endo-/lysosomes. The representative fluorescence micrographs show the results for untreated cells as control (**a**), and cells treated with nanospheres (**b**), submicrospheres (**c**), microspheres (**d**), submicrorods (**e**), and microrods (**f**).

The localization of internalized silica particles inside NR8383 cells after 24 h of exposure was also assessed by CLSM with fluorescent PEI-FITC-labeled particles and LysoTracker Red DND-99, which allowed the labeling and tracking of acidic organelles like endosomes, lysosomes, and endolysosomes. The overlays of the PEI-FITC and LysoTracker signals revealed that all particle types were mainly associated with endo- and lysosomal structures (Fig. 7). It is well accepted that internalized particles mainly end up in endolysosomal compartments^46,52,55−57^.

**Figure 8:**
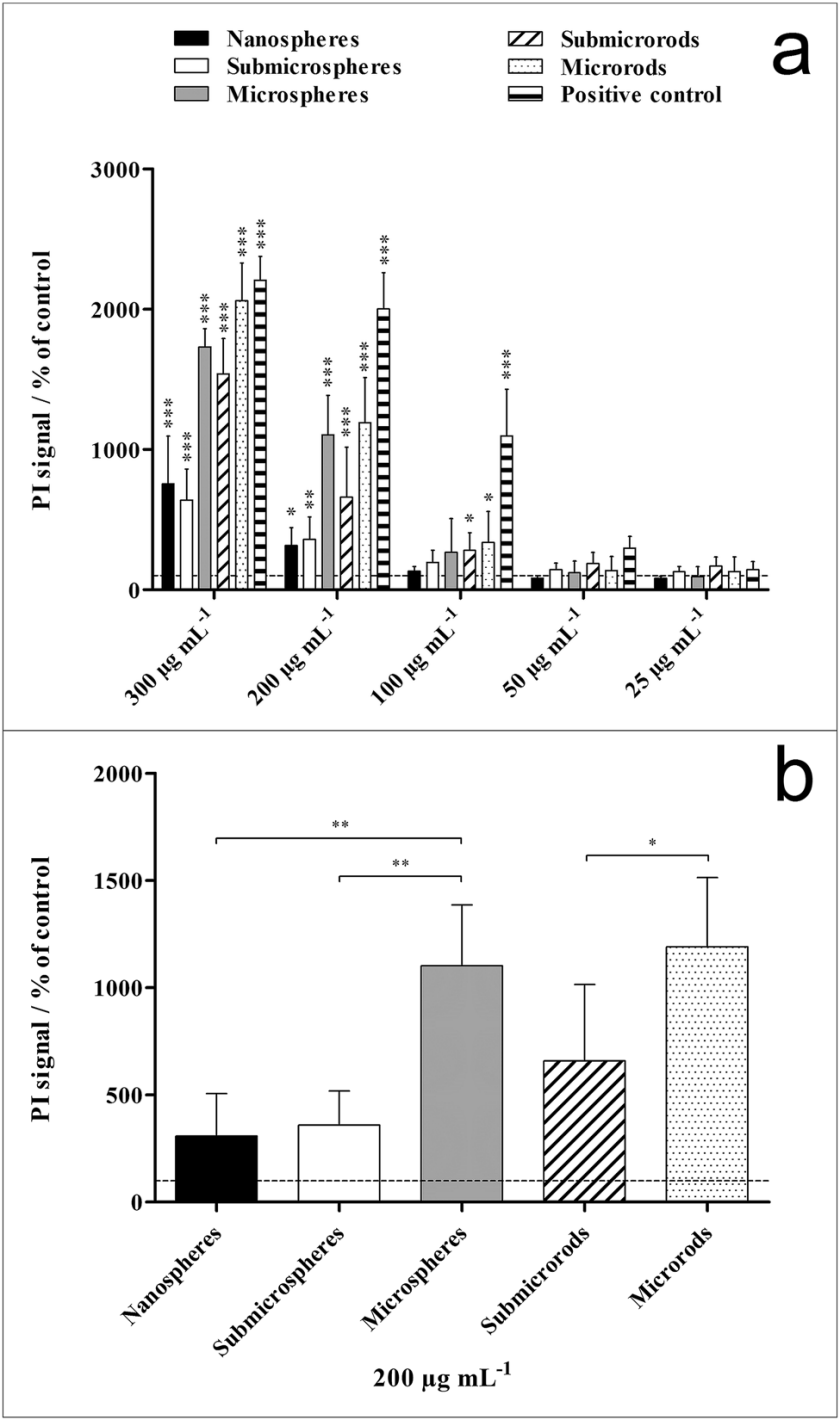
Viability of NR8383 alveolar macrophages after 16 h incubation with different unfunctionalized silica particles. The cell viability was determined by propidium iodide (PI) staining of non-viable cells and mean PI fluorescence intensity assessment by flow cytometry. Commercial silica particles served as positive control. (**a**) Comparison of the effects of different particle concentrations and morphologies on the cell viability. (**b**) Comparison of the effects of different particle morphologies on the cell viability at a particle concentration of 200 µg mL^−1^. Data are expressed as mean ± SD (*n* = 3), given as percentage of the control (100%, untreated cells). Asterisks (*) indicate significant differences in comparison to the control (**p < 0.01, ***p < 0.001).

The cytotoxic effects of the different silica particles on NR8383 cells at various particle concentrations were determined by propidium iodide (PI) staining of non-viable cells after 16 h of incubation (Fig. 8). In general, a concentration-dependent uptake of each particle type was observed (correlation coefficient > 0.86) as analyzed by flow cytometry (data not shown). A statistically significant cell toxicity was found for all silica particle morphologies (with nanospheres being the only exception) at high particle concentrations of 200 and 300 µg mL^−1^, while concentrations below 200 µg mL^−1^ had no significant effect on the cell viability except the available silica nanoparticles that served as positive control (agglomerated amorphous silica nanoparticles with an agglomerate size of 2000 ± 1000 nm)^36^. For these, a statistically significant effect on cytotoxicity was observed at 100 µg mL^−1^. Among the spherical silica particles, microspheres exhibited the highest toxicity, which was significantly enhanced compared to nanospheres and submicrospheres, indicating a size-dependent effect. Similarly, in the case of the rod-shaped particles the cell toxicity of microrods was significantly higher compared to submicrorods. In general, the cytotoxic concentrations agree with the literature on silica (nano-)particles^1,8,18^.

**Figure 9:**
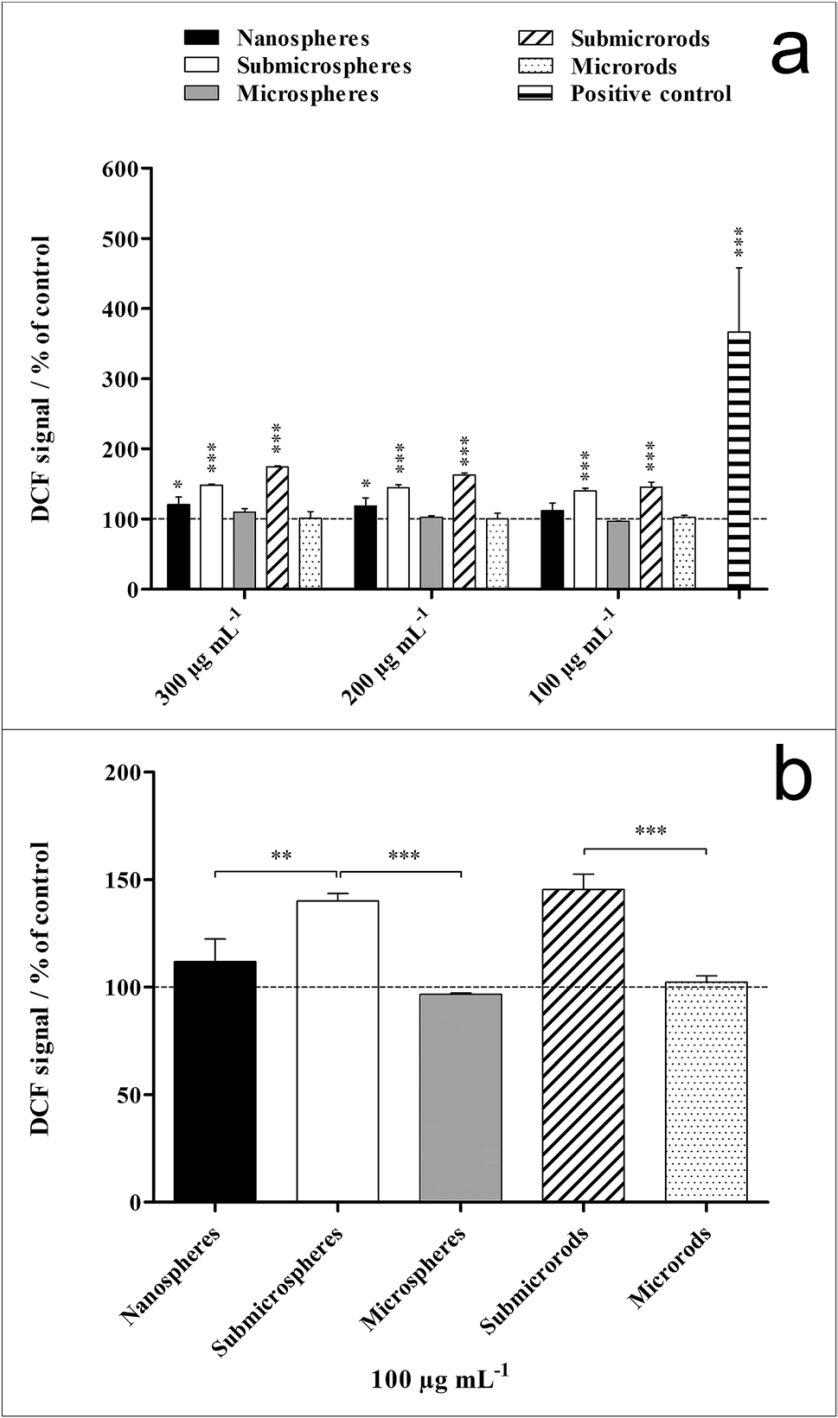
Generation of ROS in NR8383 alveolar macrophages after 2 h exposure to different unfunctionalized silica particles. ROS generation was assessed by the mean fluorescence intensity of dichlorofluorescein (DCF) using flow cytometry. A solution of 3% H_2_O_2_ served as positive control. (**a**) Comparison of the effects of different particle concentrations and morphologies on the ROS generation. (**b**) Comparison of the effects of different particle morphologies on the ROS generation at a particle concentration of 100 µg mL^−1^ (subtoxic level). Data are expressed as mean ± SD (*n* = 3), given as the percentage of the control (100%, untreated cells). Asterisks (*) indicate significant differences in comparison to the control (*p < 0.05, **p < 0.01, ***p ≤ 0.001).

Reactive oxygen species (ROS) generated by NR8383 macrophages after 2 h of exposure to silica particles of different morphologies were detected by the dichlorofluorescein (DCF) assay (Fig. 9). Significantly enhanced ROS levels in comparison to untreated cells which can be associated with cell activation were detected for silica nanospheres, submicrospheres and submicrorods, similar to the positive control (3% H_2_O_2_ served as positive control). Silica microspheres and microrods did not affect ROS production. Cell activation was most pronounced for the submicroparticles (submicrospheres and submicrorods) and significantly enhanced compared to both nano- and microparticles.

**Figure 10:**
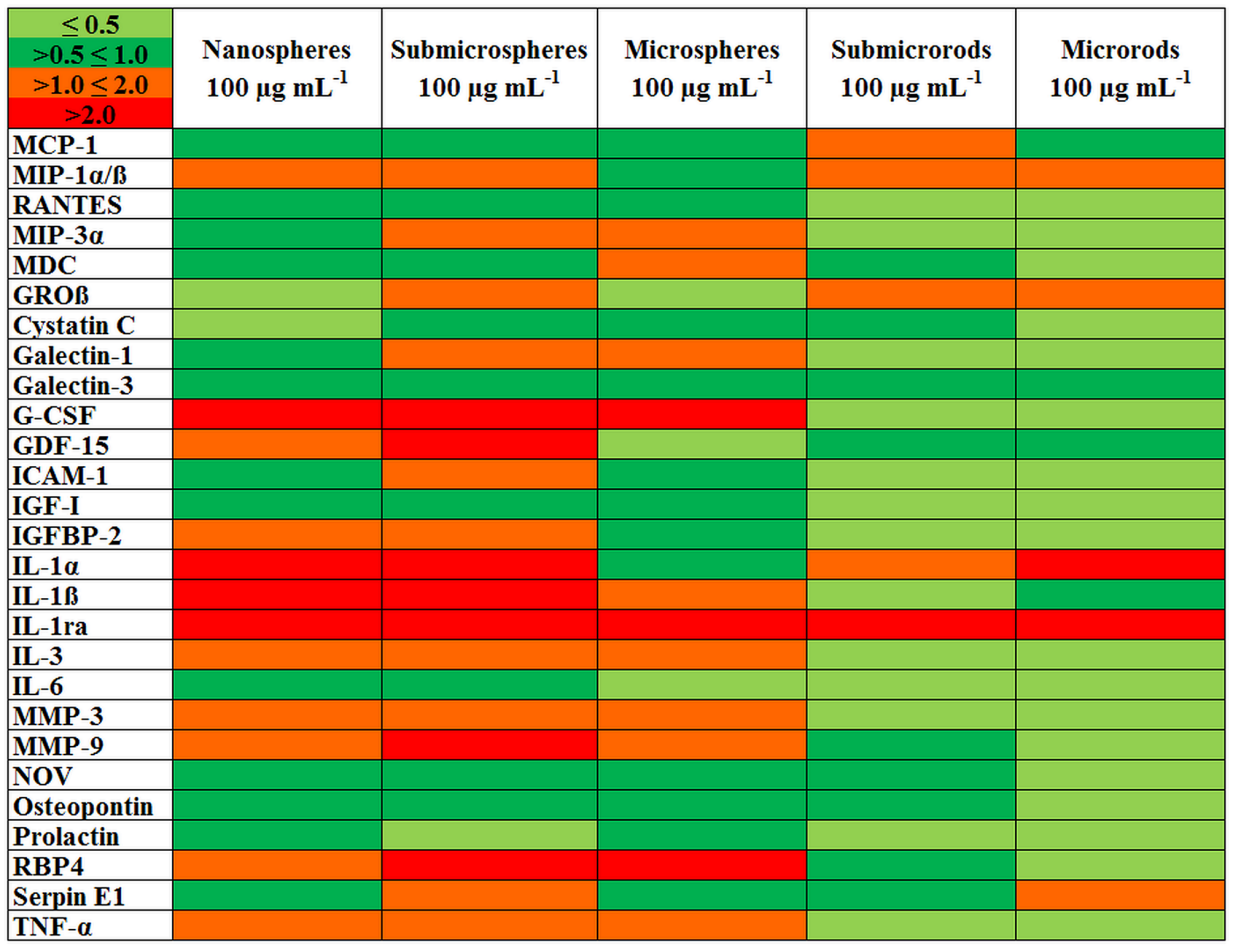
Heat map of 27 bioactive factors of the proteomic repertoire of NR8383 alveolar macrophages after 16 h of exposure to different unfunctionalized silica particles at 100 µg mL^−1^ (subtoxic level) as obtained by protein microarrays. Each row of the heat map represents the expression of one factor relative to its expression in supernatants of NR8383 cells without particle exposure (control). The green colors represent lower, the red colors represent higher expression of factors relative to the control. The color intensity scale shows fold changes at the upper left corner of the figure.

The effects of different silica particles on the expression of bioactive factors by NR8383 macrophages were investigated with cell culture supernatants after 16 h of particle exposure (100 µg mL^−1^; subtoxic level) and a chemiluminescence-based protein microarray which allowed the simultaneous detection of 79 signaling molecules like cytokines, chemokines, and growth factors. Based on the proteomic repertoire of NR8383 cells as reported by Duhamel et al.^58^, 27 of the 79 detected factors were selected for a detailed analysis. The qualitative heat map in Fig. 10 showed the relative expression levels of the selected factors.

**Figure 11:**
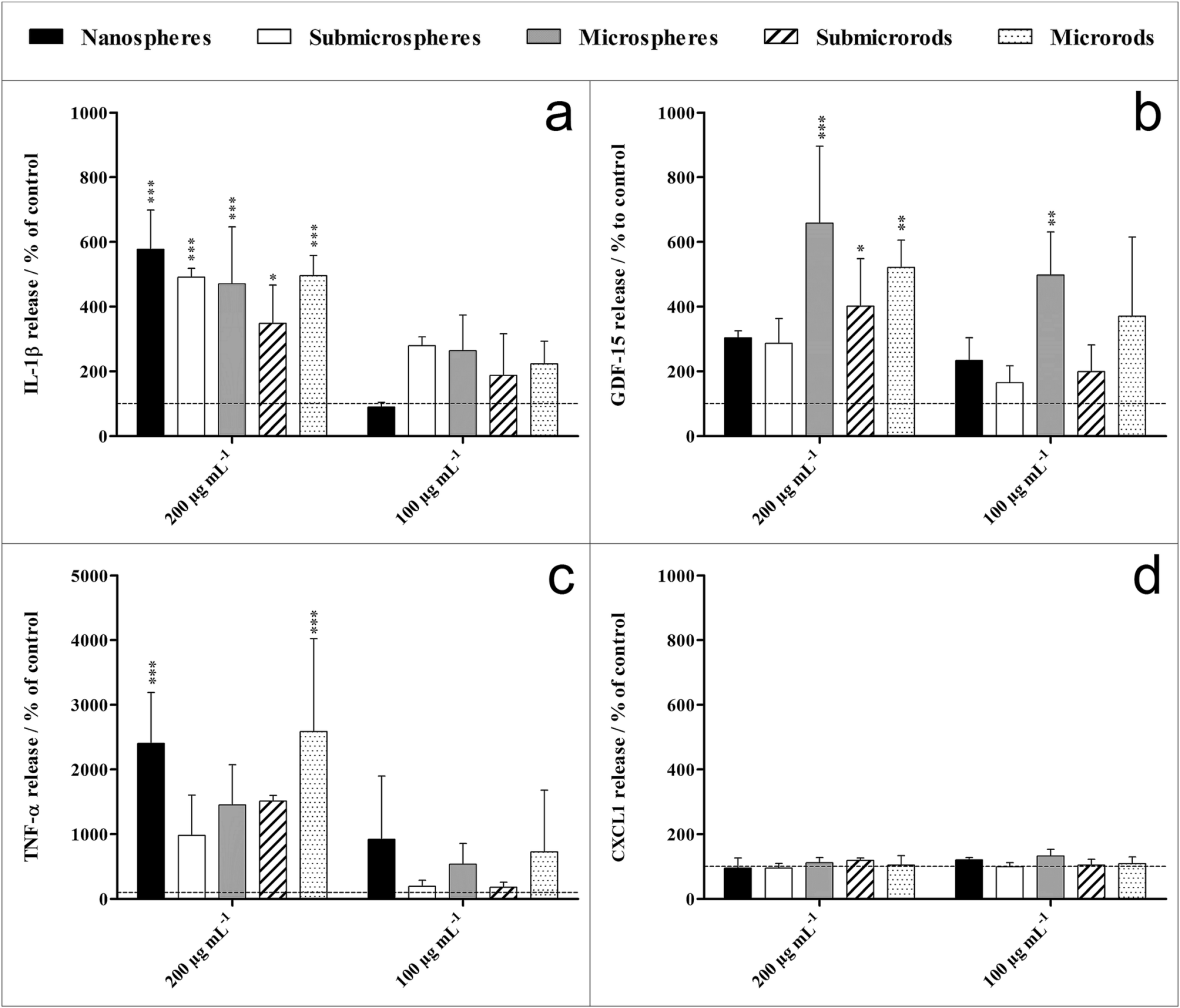
Cytokine release of NR8383 alveolar macrophages after 16 h exposure to different unfunctionalized silica particles as determined by ELISA. The released amount of IL-1β (**a**), GDF-15 (**b**), TNF-α (**c**), and CXCL1 (**d**) of untreated cells (not exposed to silica particles) was 30, 160, 2 and 14 pg mL^−1^, respectively. Data are expressed as mean ± SD (*n* = 3), given as the percentage of the control (100%, untreated cells). Asterisks (*) indicate significant differences in comparison to the control (*p < 0.05, **p < 0.01, ***p < 0.001).

The factors IL-1β, GDF-15, TNF-α, and CXCL1 were selected for a detailed quantitative analysis by Sandwich-ELISA (Fig. 11). All silica particle types significantly enhanced the release of IL-1β, compared to an untreated control at a particle concentration of 200 µg mL^−1^, indicating an inflammatory response of the NR8383 cells. At this particle concentration, the GDF-15 release was also increased in the presence of microspheres and the rod-shaped submicro- and microparticles, whereas the TNF-α release was increased only by nanospheres and microrods. However, a silica concentration of 200 µg mL^−1^ was already associated with cytotoxicity (Fig. 8). At subtoxic particles concentrations of 100 µg mL^−1^, none of the silica particles induced a significant cell activation, except for the increased GDF-15 release in the presence of microspheres. Furthermore, the expression of the chemokine CXCL1 was not affected by any particle type. In addition, a particle size-dependent effect on the activation of NR8383 was not observed in this study.

**Figure 12:**
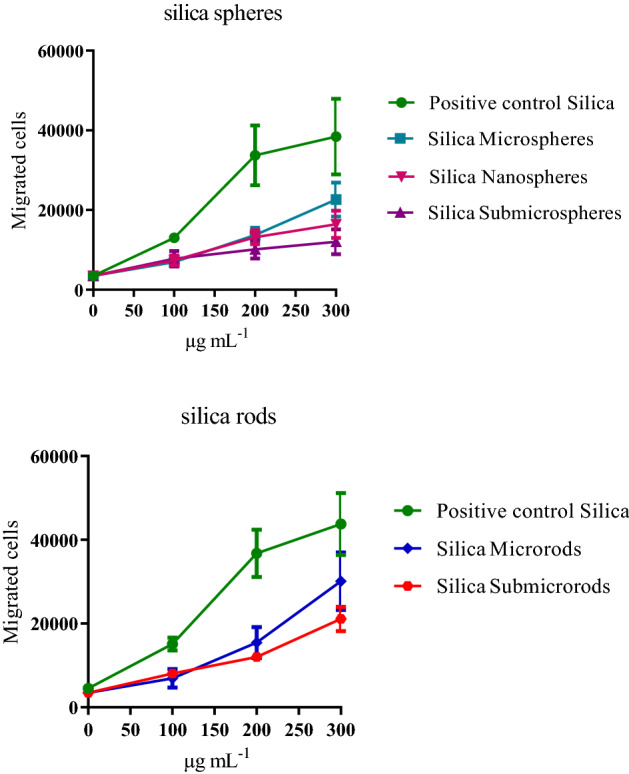
Chemotaxis (migrated cells) of the unexposed dHL-60 cells in response to NR8383 cell supernatants that were obtained from incubations with silica particles between 100 and 300 µg mL^−1^. Data are expressed as mean ± SD (*n* = 3). Commercial silica nanoparticles served as positive control. Top: Spherical silica particles with control. Bottom: Rod-shaped silica particles with control.

The potential of chemotactic factors released from NR8383 cells to recruit leukocytes (dHL-60 cells) was finally analyzed by the PICMA (Fig. 12). There were only small effects of all kinds of silica particles compared to the positive control, and there was no significant difference between the particle types. Remarkably, the commercially acquired silica particles, which served as positive control, had a significantly higher effect than the newly synthesized silica particles.

## Discussion

Usually, smaller silica nanoparticles have a higher cytotoxicity and a higher potential for the release of proinflammatory cytokines^8,27^. Chen et al. have reviewed the effect on the immune system and their interaction with the corresponding cells, including macrophages^27^. The specific surface area has been proposed as a major parameter for the cytotoxicity effects of silica nanoparticles^8,32^. The same was found for the genotoxicity with macrophages^59^.

We found only small differences between the five kinds of particles. Cytotoxic effects were only observed at high concentrations, i.e. ≥ 200 µg mL^−1^, with the microparticles being significantly more toxic (MS≈MR > SMR≈SMS≈NS) than the nanoparticles. In addition, our data on ROS formation showed that the sedimentation rate of particles (Fig. 4, particle-cell-contact) and the particle dose which is influenced by the size of the particles (the smaller the particles, the higher the dose in cell culture), probably influenced the formation of ROS. This in turn means that at a higher particle dose in cell culture (NS > SMS > MS) and a higher sedimentation rate (MS > SMS > NS) of silica particles, more ROS will be formed (Table 2). Table 3 gives a qualitative overview on the biological effects of the particles. Despite small differences, no particle type induced significant adverse effects on the cells in the subtoxic concentration range. Apparently, neither the particle size nor the particle shape play a significant role in contact with NR8383 macrophages or dHL-60 leukemia cells. However, differences in assay conditions may explain differences between published studies. It has been reported that the presence of serum within cell culture medium enhanced the polydispersity index and the hydrodynamic diameter of silica nanoparticles and led to decreased toxic effects^33^. However, is also not always clear in published studies whether the analyzed particles were agglomerated or dispersed.

**Table 3:** Summarized effects of silica particles on NR8383 alveolar macrophages.

	Uptake by cells	Relative cytotoxicity	Reactive oxygen species	Cytokine induction (overall heat map)	IL-1β	GDF-15	TNF-α	CXCL1	PICMA
Nanospheres	Yes	Low	Low	High	Low	Low	High	Low	Low
Submicrospheres	Yes	Low	Intermediate	High	High	Low	Low	Low	Low
Submicrorods	Yes	Intermediate	Intermediate	Low	High	High	Low	Low	Low
Microspheres	Yes	High	Low	Intermediate	High	High	Low	Low	Low
Microrods	Yes	High	Low	Low	High	High	High	Low	Low
Silica positive control (agglomerated)	–	High	–	–	–	–	–	–	High

All data are relative within the five particle types and the positive control.

While the biological effects of the newly synthetized silica particles differed only slightly from each other, the strongest particle-induced chemotaxis was caused by the commercially acquired silica nanoparticles, which served as positive control (Fig. 12). The characterization of these particles in a previous work^36^. showed a clear agglomeration with a particle size of 2000 ± 1000 nm that leads to a rapid sedimentation onto the cells. In the present study, the silica positive control was the only particle type that was clearly agglomerated. This observation is consistent with the hypothesis that stronger effects of nano-sized compared to micro-sized particles can be explained by the higher volume consumption of their agglomerates in the lysosomes of phagocytizing alveolar macrophages^60,61^.

The ISDD model gave the fractions of nano-, submicro-, and microspheres delivered to cells during 24 h in vitro with a medium height (liquid level) of 2.5 mm in the cell culture well. While the nanoparticles have a lower contact with the cells, both submicro- and microparticles sediment within hours. Due to the higher viscosity and density, the cell-particle contact was generally slower in medium than in pure water. Such differences in the gravitational settling of particles are a possible explanation why microparticles induced an enhanced toxic effect at high concentrations compared to the nanoparticles, consistent with the agglomerated silica positive control.

Wiemann et al. have reported extensive data on the NR8383 alveolar macrophages as in vitro model to assess the inhalation toxicity of particles^32^. In their studies, the particles had mostly sedimented onto the cells after 16 h, usually due to agglomeration. This was also the case for our particles, especially for the bigger ones (submicro and micro particles). They have classified "naked" silica particles (5 to 50 nm) as biologically active and ligand-coated silica particles as inactive. Wiemann et al. defined a threshold value of 4000 µm^2^ per cell for the distinction between active and passive materials. As shown in Table 2, these values are exceeded at 100 µg mL^−1^ for all our particles. Thus, we can conclude in line with Wiemann et al. that the observed effects were mostly due to particle overload and not to a particle-specific toxicity, in agreement with earlier opinions^1,18^. A significant cytotoxic effect of particle size or particle shape cannot be deduced from our data; the particles cause only low adverse effects.

## Conclusions

Five different types of chemically identical silica particles without surface functionalization which differ only in size and shape were prepared. All particles were readily taken up by NR8383 alveolar macrophages. Cytotoxic effects were observed at 200 µg mL^−1^ and above, where the toxicity was probably dependent on the particle-cell-contact (MS > SMS > NS) and the formation of ROS probably by the sedimentation in addition to the particle dose, which is dependent on the size of the particles (the smaller the particles the higher the particle dose). In the subtoxic concentration range (100 µg mL^−1^), all particle types induced the expression of different cytokines. A more detailed analysis of four mainly pro-inflammatory cytokines showed mixed results with no clear differences between the particles. The particle-induced cell migration assay (PICMA) confirmed that all five types of silica particles have only low pro-inflammatory potential. We conclude that at least in this size range (between 60 and 600 nm, considering all particle types) neither particle size nor particle shape are significant parameters for cell-biological effects at subtoxic concentrations. In comparison to crystalline quartz, all samples investigated here consisted of amorphous hydrated silica. The lower toxicity in comparison to quartz may be due to the hydrated state of the surface in comparison to the oxidic and more ordered crystalline surface of quartz. Further studies are necessary to elucidate the difference between amorphous and crystallize SiO_2_ species.

## Methods

### Chemicals

We used tetraethyl orthosilicate (TEOS, Alfa Aesar, 98%), aqueous ammonia (NH_3_, 25–30%, Carl Roth), ethanol (*p.a*., Fisher Chemicals), cetyltrimethylammonium bromide (CTAB, Sigma-Aldrich, 99%), branched polyethyleneimine (PEI, *M*_w_ = 25,000 g mol^−1^, Sigma-Aldrich), and fluorescein-labeled polyethyleneimine (PEI-FITC, *M*_w_ = 25,000 g mol^−1^, Sigma-Aldrich) as obtained. All syntheses were carried out in ultrapure water (Purelab ultra instrument from ELGA). Prior to the syntheses, the glassware was cleaned first with boiling *aqua regia* and then twice with boiling water. The particles were isolated by centrifugation with a Heraeus Fresco 21 centrifuge (Thermo Scientific). All particles were endotoxin-free as analyzed by the Limulus Amebocyte Lysate test with a detection limit of < 0.01 EU mL^−1^ (Endosafe nexgen-PTS, Charles River)^62^.

### Instruments

For dynamic light scattering for particle size analysis and zeta potential determination, we used a Malvern Zetasizer Nano ZS ZEN 3600 instrument (Malvern Panalytical Ltd.; 25 °C, laser wavelength 633 nm), operating at a fixed angle of 173° in backward scattering mode. The peak profile of the size distribution was analyzed by a log-normal distribution fit. The average particle diameter is given as mean value of the maximum of the size distribution from the log-normal distribution fit analysis and the empirical standard deviation as given by the instrument software.

Ultraviolet–visible spectroscopy (UV/vis) was carried out with a Varian Cary 300 instrument (Agilent Technologies, Inc.) in Suprasil micro quartz cuvettes with a sample volume of 750 µL after dilution and background correction.

Fourier-transformed infrared spectroscopy (FT-IR) was performed with an Attenuated Total Reflection (ATR) Bruker Alpha Platinum FT-IR spectrometer. FT-IR spectra of dried silica particles and pure CTAB were obtained by pressing them onto a diamond plate.

Confocal laser scanning microscopy was performed with a Zeiss LSM 700 instrument (Carl Zeiss Microscopy GmbH, Jena, Germany). Fluorescence images were taken (Zeiss LSM 700 microscope and Zen 2010 software) and digitally processed using Adobe Photoshop 7.

Flow cytometric analyses were carried out with an FACSCalibur flow cytometer (BD Bioscience, Heidelberg, Germany). For each measurement, 10,000 cells were analyzed and the data were quantified with the CELLQuest 1.2.2 software (BD Biosciences).

Scanning electron microscopy of the particles was performed with a microscope type FEI ESEM Quanta 400 FEG (ThermoFisher). Prior to the investigation, the samples were sputter-coated with a thin conductive AuPd 80:20 layer.

For X-ray powder diffraction, we isolated the nanoparticle dispersion, shock-froze it with liquid nitrogen, and lyophilized it at 0.31 mbar and − 10 °C in a Christ Alpha 2-4 LSC freeze-dryer. X-ray powder diffraction was carried out with a Bruker D8 ADVANCE (Bruker Corp.) diffractometer operating in Bragg–Brentano geometry with Cu Kα radiation (*λ* = 1.54 Å, 40 kV and 40 mA). As sample holder, a single-crystalline silicon sample holder with a crystallographic (911) plane to minimize background scattering was used.

The FIB system (type FEI Helios G4 CX) consisted of an electron column and an ion column. We used gallium as liquid metal ion source to produce cross-sections of cells at an acceleration voltage of 30 kV together with a stepwise current reduction from 22 to 2.7 nA. SEM images were recorded at an acceleration voltage of 5 kV. TEM imaging and EDX analysis were performed with a FEI Tecnai F20 instrument.

### Synthesis of silica particles

Silica nanospheres, submicrospheres, and microspheres were obtained by a modified Stöber process, following the protocols described in the literature with some modifications^39,40^. For nanospheres, ammonia (13.2 mL, 25%) was added to ethanol (200 mL) at 68 °C under continuous stirring. After 30 min, TEOS (5.2 mL) was added to the mixture. The reaction was continued with stirring for 4 h at 68 °C, followed by stirring at room temperature for 12 h. The nanoparticles were isolated by centrifugation and redispersion in water (3,500 rpm, 90 min, three times), redispersed in ethanol, and dried in air at 200 °C for 3 h. For submicrospheres, the procedure consisted of mixing water (6.1 mL) and ammonia (2.8 mL, 30%) at 47 °C in ethanol (50 mL), followed by the addition of TEOS (6.2 mL) and stirring for 4 h. After 4 h, the reaction mixture was cooled to room temperature and stirred for another 16 h. The particles were isolated and purified as described above. Microspheres were synthesized by the following procedure: Ammonia (4.2 mL, 30%) and water (4.8 mL) were added to ethanol (50 mL) at room temperature under continuous stirring for 20 min. Then, TEOS (4.9 mL) was added and the mixture was stirred for another 16 h at room temperature. After synthesis, silica microspheres were collected by centrifugation (3,500 rpm, 30 min) and washed with ethanol several times.

Silica submicrorods and microrods were synthesized by a modified Stöber process according to Ref.^63^ with a modified molar ratios of the reagents. For the synthesis of silica submicrorods, CTAB (0.12 g) was dissolved in water (30 mL) and stirred at 30 °C for 30 min. After cooling the solution to room temperature, ammonia (0.3 mL, 25%) was added to the CTAB solution and stirred for 20 min. Then, TEOS (0.25 mL) was added and the reaction mixture was stirred for 1 h. After 7 min, the originally clear solution had assumed a turbid white color. For the synthesis of silica microrods, CTAB (0.3 g) was completely dissolved in water (40 mL) and stirred at 30 °C for 30 min. Ammonia (1 mL, 30%) was added. After 20 min, TEOS (1.8 mL) was added and stirring of the reaction mixture was continued at room temperature for 1 h. Silica submicrorods and microrods were collected by centrifugation (3500 rpm, 30 min) and washed with ethanol several times. In the next step, the particles were was redispersed in ethanol and heated at 60 °C for 6 h in an ethanolic HCl solution (EtOH:HCl = 0.99:0.01 *v*/*v*) to remove the organic surfactant (CTAB). The silica particles were purified by triple centrifugation (3500 rpm, 30 min) in ethanol and dried at 200 °C for 3 h. The quantitative absence of CTAB was shown by IR spectroscopy (Fig. 3).

The number of particles in 1 g solid was then computed from the average particle mass for spheres and rods:

$$ m_{{{\text{sphere}}}} = \frac{4}{3}\pi r^{3} \rho $$

$$ m_{{{\text{rod}}}} = \pi r^{2} L \rho $$

with *r* the particle radius and *L* the particle length, both obtained by SEM (Table 1), and *ρ* the density of silica (2000 kg m^3^)^63^. The specific surface area of particles (m^2^ g^−1^) was calculated as follows:

$$ S_{{{\text{spheres}}}} = \, 4\pi r^{2} N_{{{\text{particles}}\,{\text{per}}\,1\,{\text{g}}}} $$

$$ S_{{{\text{rods}}}} = 2\pi r^{2 } + 2\pi rLN_{{{\text{particles}}\,{\text{per}}\,1\,{\text{g}}}} . $$

### Functionalization of silica particles with FITC-labeled polyethyleneimine (PEI-FITC)

The dried silica particles were surface-functionalized with FITC-labeled polyethyleneimine (4 µL, 1 g L^−1^) by stirring a particle dispersion in ethanol (2 g L^−1^) overnight at room temperature under light exclusion. Purification of the particles was performed by double centrifugation (AMICON Ultra-15 centrifugal filters, MWCO 3 kDa, 3,500 rpm, 45 min) in ultrapure water, followed by freeze-drying of the particles. The fluorescently labeled silica particles were freeze-dried with an alpha 2-4 LSC instrument (Christ).

### Particle storage and treatment

The unfunctionalized particles were stored after synthesis at 4 °C in the dark under argon atmosphere. The PEI-labeled particles were freeze-dried after synthesis. Stock dispersions were prepared in water at a particle concentration of 1 g L^−1^. Before adding to the cell culture media, the stock dispersions were diluted to 5 to 80 mg L^−1^. Each dispersion was thoroughly vortexed after dilution and treated in an ultrasonic bath (Elma Sonic S10, 30 W, 50/60 Hz, 450 mL volume) for 5 min to obtain well-dispersed particles. All particle preparations were vortexed again immediately before application.

### Cell culture

The biological characterization of the particles was performed with the cell line NR8383 (rat alveolar macrophages, LGC Standards GmbH, Wesel, Germany). The cells were cultivated with Ham's F-12 medium containing 15% fetal calf serum (FCS, GIBCO, Invitrogen, Karlsruhe, Germany) in 175 cm^2^ cell culture flasks (BD Falcon, Becton Dickinson GmbH, Heidelberg, Germany) at standard cell culture conditions (humidified atmosphere, 37 °C, 5% CO_2_). NR8383 cells were partly adherent and partly non-adherent. The ratio between adherent and non-adherent cells was about 1:1. For cell experiments, adherent cells were detached from the cell culture flasks with a TPP cell scraper (TPP Techno Plastic Products AG, Trasadingen, Switzerland), subsequently combined with non-adherent cells and seeded into 24-well cell culture plates (BD Falcon) at a concentration of 2.4 × 10^5^ cells cm^−2^.

### Particle uptake analyzed by FIB/SEM, TEM/EDX, and CLSM

We studied the uptake of silica particles by NR8383 cells by cross-sectional analysis of single cells, accomplished by focused ion beam milling (FIB) in combination with scanning electron microscopy (SEM), transmission electron microscopy (TEM), and energy-dispersive X-ray spectroscopy (EDX). After the fabrication of thin lamellae by FIB, the samples were imaged by TEM in combination with EDX for a detailed analysis.

To analyze the uptake of nanoparticles, NR8383 were exposed to 100 µg mL^−1^ of unfunctionalized silica particles for 24 h under cell culture conditions, detached from the culture plate as described above, and seeded on a titanium carrier plate (25 mm^2^). The cells were washed with PBS (GIBCO, Invitrogen) after 3 h of adherence under cell culture conditions and fixed with 3.7% glutaraldehyde (Sigma-Aldrich, Taufkirchen, Germany) in PBS (*v*/*v*). The cells were then washed again with PBS and dehydrated in an ascending ethanol row (50%, 70%, 90%, and 100%, 5 min each) according to standard procedures. After the dehydration step, the cell samples were mounted on SEM carriers and finally sputter-coated with 15 nm gold–palladium layer (K500X, Quorum Technologies Ltd., Ashford Kent, United Kingdom). Before the FIB milling, the samples were furthermore sputter-coated with a thin layer of carbon to protect the cells them from ion contamination and damage during the FIB procedure. The carbon layer was deposited with a gas injection system, delivering gaseous naphthalene that adsorbed to the sample surface. The naphthalene molecules were then decomposed by the electron or ion beam in a defined rectangular position. There, the beam was scanned over the sample surface. The deposited carbon layer remained on the surface, and gaseous decomposition products were removed by a vacuum pump.

Confocal laser scanning microscopy (CLSM) was used for detailed co-localization studies. The NR8383 cells were incubated with 100 µg mL^−1^ of PEI-FITC-labeled silica particles for 24 h under standard cell culture conditions and then stained with 50 nM of a specific endo/lysosomal fluorescent probe (LysoTracker Red DND-99, Invitrogen) in pure Ham’s F-12 medium for 30 min at 37 °C. The cells were analyzed by CLSM after three rinses with Ham’s F-12 medium.

### Cytotoxicity assay

The cytotoxicity of silica particles was measured and quantified by flow cytometry. As positive control, silica nanoparticles were used (CAS No. 7631-86-9, Lot MKBF2889V, 99.5%, 10–20 nm) (Sigma-Aldrich, Steinheim, Germany). The particles were previously characterized in detail and consisted of agglomerated X-ray amorphous silica particles with a primary particle size of about 49 nm and an agglomerate size of about 2 µm^36^.

After 16 h of exposure to various silica concentrations (300, 200, 100, 50, 25 µg mL^−1^), adherent cells were detached from the cell culture plates with a TPP cell scraper, combined with non-adherent cells and transferred to 5 mL tubes (BD Biosciences). Non-viable cells were labeled with 50 µg mL^−1^ propidium iodide (PI, Sigma-Aldrich) for 10 min at room temperature, and the number of non-viable cells (PI positive) was analyzed by flow cytometry.

### Generation of reactive oxygen species (ROS)

For the analysis of ROS formation induced by the exposure of NR8383 cells to silica particles, a DCF assay was performed by flow cytometry. The cells were incubated with different concentrations of silica particles (300, 200, 100 µg mL^−1^) for 2 h under cell culture conditions. A solution of 3% hydrogen peroxide (H_2_O_2,_ Herbeta Arzneimittel, Berlin, Germany) served as positive control. The cells were incubated with 3% H_2_O_2_ for 30 min under cell culture conditions.

Subsequently, 20 µM of the cell-permeable ROS indicator 2′,7′-dichlorodihydrofluorescein diacetate (H_2_DCFDA, Thermo Fisher Scientific, Waltham, USA) was added, and the cells were incubated at 37 °C for 30 min. The non-fluorescent H_2_DCFDA diffuses into cells, where it is deacetylated by intracellular esterases and converted to the highly fluorescent 2′,7′-dichlorofluorescein (DCF) form by oxidation. For discrimination between viable and non-viable cells, an additional PI staining was performed.

### Protein microarray and ELISA

After incubation of NR8383 cells with different silica particles (100 µg mL^−1^) for 16 h, the supernatants were centrifuged at 300 g for 10 min and stored at -20 °C until the microarray analysis (Profiler Array Rat XL Cytokine Array Kit, Bio-Techne GmbH, Wiesbaden-Nordenstadt, Germany). The assay detected 79 different cytokines, growth factors and other mediators and enabled a semiquantitative analysis. The membrane-based sandwich immunoarray consists of a nitrocellulose membrane on which the captured antibodies are spotted as duplicated dots. Target proteins present in the sample bind to the capture antibodies and are detected with biotinylated detection antibodies and then visualized with chemiluminescent detection reagents. For analysis, the manufacturer's instructions were observed and chemiluminescence signals were detected and quantified by a microarray imager and the ImageQuantTL software (Amersham Imager 600 RGB, GE Healthcare Bio-Science, Uppsala, Sweden). For subsequent detailed analysis using Sandwich-ELISA Kits (R&D Systems Quantikine, Bio-Techne GmbH, Wiesbaden, Germany), 27 factors were selected based on the proteomic repertoire of NR8383 cells, as reported by Duhamel et al.^58^.

### Particle induced cell migration assay (PICMA)

NR8383 cells were cultivated at 37 °C, 100% humidity, and 5% CO_2_ in Ham's F-12 + 15% FCS (fetal calf serum, Biochrom KG, Berlin, Germany), 2 mM L-glutamine, 100 µg mL^−1^ penicillin, and 100 U mL^−1^ streptomycin. Approximately 3 × 10^6^ cells were seeded in 25 mL (175 cm^2^) medium.

HL-60 cells were obtained from DSMZ (Braunschweig, Germany). For the investigation of chemotaxis, we used *trans*-retinal differentiated HL-60 cells (dHL-60). The HL-60 cells were cultivated in RPMI 1640 medium (Biochrom KG) + 10% FSC, 2 mM L-glutamine, 100 µg mL^−1^ penicillin, 100 U mL^−1^ streptomycin, and 1 μM *trans*-retinoic acid at 37 °C, 100% humidity and 5% CO_2_ for three days^64^. In general, the dHL-60 cells grow adherent to standard culture dishes.

For the PICMA, NR8383 rat macrophages (3∙10^6^ cells mL^−1^) were suspended with a vortex in 1 mL Ham's F-12 medium + 15% FCS, 2 mM L-glutamine, 100 µg mL^−1^ penicillin, and 100 U mL^−1^ streptomycin and seeded in 12.5 cm^2^ cell culture flasks at a final volume of 3 mL (2.4∙10^5^ cells cm^−2^). The assay can also be performed in a smaller volume at constant surface volume ratio.

A sample in which cells without particles were incubated served as negative control. The subsequent experiments were repeated up to concentrations which gave the maximum induction of chemotaxis. Incubation of the NR8383 cells with the particles was performed at 37 °C, 100% humidity, and 5% CO_2_ for 16 h. Thereafter, the cells were removed by centrifugation with 300*g* for 5 min and the particles were removed by centrifugation with 15,000*g* for 10 min at room temperature. The supernatants were immediately used for the migration tests.

Cell migration was investigated according to Boyden^65^. with the modifications described by Westphal et al.^36^ and Schremmer et al.^35^, applying exclusively permanent cell lines: 200,000 unchallenged dHL-60 cells were added to 200 µL RPMI 1640 medium without FCS and seeded in each plate. A well insert (THINCERT, 3 µm pore size, Greiner bio-one, Frickenhausen, Germany) was placed into the cavities of 24 black well plates (Krystal, Dunn Labortechnik, Asbach, Germany). 500 µL of the supernatants of the particle-incubated NR8383 cells were added to the lower chamber. Migration of dHL-60 cells across the membrane was performed at 37 °C, 100% humidity, and 5% CO_2_ for 24 h. For calibration, 0 to 100,000 HL-60 cells were seeded directly into four-plate wells that were left without inserts.

Staining of migrated cells and of the calibration cells was performed with Calcein-AM for 60 min at 37 °C, 5% CO_2_, and 100% humidity by adding 500 µL Calcein-AM in the plate wells (> 90% HPLC, Sigma-Aldrich, Steinheim, Germany). Calcein-AM was delivered as 4 mM solution in DMSO, stored in aliquots at − 18 °C, and diluted to a final concentration of 4 µM in PBS.

The cell suspensions were removed from the plate wells and collected by centrifugation with 300 g for 5 min at room temperature. 850 µL of the supernatant were discarded while the cells were resuspended in the remaining volume of 150 µL. In addition, the adherent cells at the outside of the inserts were detached by adding 500 µL trypsin/EDTA (0.05%/0.02%, Biochrom) for 10 min at 37 °C, 5% CO_2_, and 100% humidity. Subsequently, the inserts were removed from the plate wells. Then, 150 µL of the collected cells were added into the plate wells that contained the 500 µL of the trypsin/EDTA-detached cells. The cell count was determined by fluorescence spectroscopy at 490/520 nm and calculated from the cell calibration (SpectraMax M3, Molecular Devices, Sunnyvale, USA).

Acceptance criteria for a valid test were positive (nanosized silica) and negative controls within the range of the established controls in our laboratory. A positive response was defined as a dose-dependent increase of cell migration of at least two consecutive concentrations with a maximum exceeding the base rate by at least two times at the highest concentration^36^.

The EC_50_ was calculated separately for each compound using nonlinear regression and the GraphPad Prism 8 software.

As reference, we used a silica reference sample (CAS No. 7631-86-9, Lot MKBF2889V, 99.5%, 10–20 nm) (Sigma-Aldrich, Steinheim, Germany). The particles were previously characterized in detail and consisted of agglomerated X-ray amorphous silica particles with a primary particle size of about 49 nm and an agglomerate size of about 2 µm^36^.

### Statistical analysis

Data are expressed as the mean ± SD (*n* = 3) and given as the percentage of the control (cells not exposed to particles). For statistical evaluation, one-way analysis of variance (ANOVA) with Dunett's Multiple Comparison Test was applied using the GraphPad Prism software (GraphPad Software, Inc., CA, USA), while *p* values ≤ 0.05 were considered as statistically significant.
